# Healthcare Workers’ Attitudes toward Influenza Vaccination: A Behaviour and Social Drivers Survey

**DOI:** 10.3390/vaccines11010143

**Published:** 2023-01-09

**Authors:** Binshan Jiang, Yanlin Cao, Jie Qian, Mingyue Jiang, Qiangru Huang, Yanxia Sun, Peixi Dai, Heya Yi, Run Zhang, Lili Xu, Jiandong Zheng, Weizhong Yang, Luzhao Feng

**Affiliations:** 1 School of Population Medicine and Public Health, Chinese Academy of Medical Sciences & Peking Union Medical College, Beijing 100730, China; 2 Division of Infectious Disease, Chinese Center for Disease Control and Prevention, Beijing 102206, China; 3 Department of International Affairs, Chinese Preventive Medicine Association, Beijing 100062, China; 4 “Breath Circles” Network Platform, Beijing 100026, China; 5 Institute for Non-Communicable Disease Control and Prevention, Qinghai Provincial Center for Disease Control and Prevention, Xining 810007, China

**Keywords:** influenza vaccine, healthcare workers, influenza vaccine hesitancy, behaviour and social drivers, recommendation

## Abstract

This study aimed to understand the intention and correlation of receiving and recommending influenza vaccine (IV) among healthcare workers (HCWs) in China during the 2022/2023 season using the behavior and social drivers (BeSD) tools. A self-administered electronic survey collected 17,832 participants on a media platform. We investigated the willingness of IV and used multivariate logistic regression analysis to explore its associated factors. The average scores of the 3Cs’ model were compared by multiple comparisons. We also explored the factors that potentially correlated with recommendation willingness by partial regression. The willingness of IV was 74.89% among HCWs, and 82.58% of the participants were likely to recommend it to others during this season. Thinking and feeling was the strongest domain independently associated with willingness. All domains in BeSD were significantly different between the hesitancy and acceptance groups. Central factors in the 3Cs model were significantly different among groups (*p* < 0.01). HCWs’ willingness to IV recommendation was influenced by their ability to answer related questions (r  =  0.187, *p*  <  0.001) after controlling for their IV willingness and perceived risk. HCWs’ attitudes towards IV affect their vaccination and recommendation. The BeSD framework revealed the drivers during the decision-making process. Further study should classify the causes in detail to refine HCWs’ education.

## 1. Introduction

Annual influenza vaccination for healthcare workers (HCWs) has been recommended as a top priority in many countries, including China, since 2018 because of their occupational health [1,2]. Modest evidence has shown that indirect protection modifies outbreaks by vaccinating HCWs to protect them and patients from nosocomial infection [3,4]. There is a consensus regarding the prediction of the spread of influenza in the coming winter and spring since the surge of influenza activity early this year surpassed the spread of the SARS-CoV-2 virus globally and attained a peak in Southern China [5]. However, HCWs influenza vaccination coverage (IVC) had actually fallen partially due to inadequate implementation of free vaccination and resource shortage during COVID-19 [6]. Suppression of influenza spread and waning immunity in populations affected by the COVID-19 pandemic interventions would aggravate the disease burden, especially among HCWs at an increased risk due to occupational exposure this year, and would even have the supra-seasonal pattern potential [7,8]. Thus, the importance of the influenza vaccine (IV) cannot be overemphasized. As the interventions against the spread of COVID-19 are lifted, other respiratory infectious diseases will return, putting a potentially overwhelming strain on medical resources and exposing HCWs to risks. IV protects HCWs’ occupational health and reduces transmission to susceptible patients. Frontline HCWs will be crucial in the height of influenza-without them, patient care will suffer, and the healthcare systems will be stretched. As the IV campaigns are about to be set out in September to October in China, HCWs’ attitudes toward this season’s vaccination remain obscure.

The World Health Organization (WHO) released the first position paper to investigate the behaviour and social drivers (BeSD) influencing the decision-making process during vaccination [9]. The BeSD framework proposes that constructs from four domains can influence vaccine uptake: thinking and feeling, social processes, motivation, and practical issues. Each domain may determine individuals’ behaviour independently or mutually. It advocated countries devote immunization practices to consider broader behaviour and social drivers. Meanwhile, it appealed to evidence from low- and middle-income countries to contribute to the further development of the BeSD survey toolkits. Before the BeSD, the 3Cs’ model developed by the WHO had been widely used to differentiate the drivers of vaccine hesitancy [10]. Thus, our study intended to take a new look at HCWs’ influenza vaccination-related behaviour following the BeSD framework and 3Cs’ model. Since the single most potent intervention for increasing IV uptake is a provider recommendation which is strongly associated with HCWs’ vaccination behaviour [11]. Understanding the driving forces that contribute to identifying essential changeable factors that impede vaccination among HCWs, and designing vaccine-related research and campaign programs to change their behaviour could improve IV coverage generally in China. Thus, the aims of the study were to assess the willingness of HCWs to vaccinate against influenza and their attitude towards recommending it to others in the coming season. HCWs were chosen because they were about to face a high risk of contracting influenza and were a priority group for influenza vaccination [12].

## 2. Materials and Methods

### 2.1. Patient and Public Involvement

As this study focussed on HCWs, patients or the general public were not involved in the study design or outcome measures. However, medical students were involved with the target group in the piloting of the survey, which intended to test the logical and statement of the questionnaire.

### 2.2. Participants and Data Collection

An online survey was conducted at the beginning of the IV campaign in mid-September 2022 on the *Breath Circles* forum, a platform based on WeChat, mainly targeting an audience comprising respiratory disease medical care practitioners. A link to the self-designed questionnaire was posted on the *Breath Circles* forum so HCWs who received the invitation could forward it to their colleagues. The sample size was estimated by the formula $N=\mu_{\alpha }^{2}\times p\times \left(1-p\right)/\delta^{2}$, based on 5% type one error, the rate of HCWs’ willingness to take the influenza vaccine (*p*) = 30%, and maximum permissible error (δ) = 0.1 × *p*. We estimated a sample size of 897 participants from each type of workplace. Considering the potential invalid response, the sample size was 987 for each type after increasing by 10%. The survey was allowed to enlarge the sample size in financial conditions permitting. Informed consent was obtained at the beginning of the survey; each participant could respond once. All data were de-identified, and the participants were anonymous. Those who failed the attention check answered all questions with the same responses and whose completion time was less than 0.5% (32 s) or more than 99.5% (832 s) were disqualified.

### 2.3. Measurement and Variables

The primary outcomes were assessed by two questions: (a) the willingness to be vaccinated in the 2022/2023 season, and (b) the willingness to recommend the vaccines to others, using a 5-point scale with options as “definitely not,” “probably not,” “not sure,” “probably yes,” and “definitely yes.” The willingness was categorized into three groups to understand the hesitancy for influenza vaccine and recommendation among HCWs: high acceptance, moderate acceptance, and hesitancy (those without positive attitude) [13]. 

The survey included five sets of independent variables: demographic characteristics of participants, including gender, working area, type of health institution, and professional title; IV information, including vaccination record and vaccination place in the previous season; the BeSD of IV uptake; the 3Cs’ variables; and the factors that impede HCWs recommendation measured using multiple choice questions with an order. The information on BeSD of IV uptake was collected by adopting the health worker items of the COVID-19 vaccination survey provided by WHO as a reference. The acceptance of the influenza vaccine was counted by each BeSD item. For the 3Cs’ variables, statements of confidence, convenience, and complacency, adopted from previous studies were included [6], but the average scores of each factor in the 3Cs’ model were calculated by a 5-point Likert scale ranging from 1  =  “not at all” to 5  =  “fully compatible/right/follow” to identify factors influencing vaccine hesitancy. 

## 3. Statistical Analysis

Data were collected using Microsoft Excel (version 2019, Redmond, WA, USA) and analyzed with SPSS version 26 (Armonk, NY, USA) and RStudio (version 1.4.1, RStudio Inc., Boston, MA, USA). Absolute and relative frequencies were calculated for categorical variables, and the chi-square test was used for comparisons. Univariate and multivariate logistic regressions were performed to examine the predictive indicators of IV intention. The Likert scale in the 3Cs’ model was used as continuous variables, and the averages of each factor were compared by analysis of variance and Fisher’s least significant test. Partial correlation analysis was conducted for recommendation willingness and the ability to answer patient questions, controlling for influenza vaccine hesitancy (IVH), perceived risk of influenza, and last year’s IV behaviour. The cumulative percentages for multiple-choice questions were calculated and arranged in order using weighted scores. Two-sided *p*-values < 0.05 indicated a statistically significant difference.

## 4. Results

In all, 21,470 HCWs participated: 17,832 (83.06%) valid responses were included in the analysis, 13,533 (75.89%) of the participants were from medical institutions, 5979 (44.18%, 5979/13,533) were working at tertiary hospitals. The majority were men (n = 10,042, 56.31%) and from high-economic cities in China (n = 11,787, 66.10%). The characteristics of the participants are presented in Table 1.

## 5. Attitudes and Behaviour of HCWs towards Influenza Vaccine

Among the 17,832 participants, 13,355 (74.89%) were favourable to vaccination in this season, and 14,726 (82.58%) showed their intention to certainly (n = 8562) or probably (n = 6164) recommending IV to others regardless of their IV willingness for own vaccination. 

## 6. Main Drivers of Influenza Vaccine Hesitancy by the BeSD Survey

**Figure 1:**
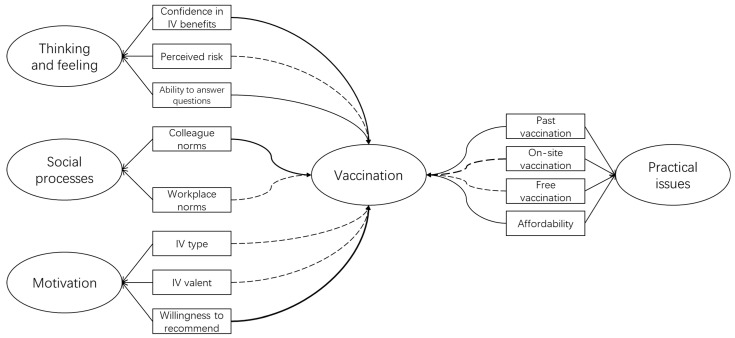
Behaviour and social drivers of influenza vaccination. Solid lines showed each level was significantly influenced by Chinese healthcare workers influenza vaccination, while broken lines showed healthcare work with some levels of the item were significantly affected when taking the high acceptance group as a reference. The thickness of lines was used to call attention to the drivers that matter most according to adjust odds ratio by multivariable logistic regression analysis. Significant refers to the difference among all levels of the item reached statistically significant in the logistic regression analysis; partially significant refers to not all levels of the item being statistically significant. IV: Influenza vaccine.

The multivariate logistic regression analysis, with high acceptance as the reference, showed that almost all the 12 items adopted from the BeSD survey had a significant influence on the IV hesitancy and the extent of acceptance (Figure 1). Especially, thinking and feeling was the strongest domain independently associated with willingness (Table 2 about here): different degrees of confidence in IV benefits increased the IVH by approximately 1.97 to 4.69 times and decreased the acceptance intensity from 2.27 to 7.26 times. The self-evaluated ability to answer questions with confidence was associated with positive attitudes adjusted odds ratio (aOR) versus “very confident” ranging from 1.44–2.13 and 1.46–1.99 in hesitancy and moderate acceptance groups, respectively). Intriguingly, the number of HCWs who were inclined to refuse or delay receiving the vaccine was less among those who lacked the sense of influenza risk (aOR and 95% CI versus “very concerned”: “not at all concerned” 0.24 (0.19–0.30), “a little concerned” 0.36 (0.29–0.45)). HCWs who were undecided or who refused to become vaccinated more often lacked colleague norms (aOR versus have colleague norms: 1.57, 95%CI: 1.39–1.78), were employed in workplaces without a vaccination-supportive attitude or policy (aOR versus requirement: 1.90, 95%CI: 1.00–1.42), were hesitant to recommend (aOR versus high willingness: 5.31, 95%CI: 4.60–6.13), failed to be vaccinated in the last season (aOR versus vaccinated: 1.28, 95%CI: 1.15–1.44), were not sure about on-site vaccination (aOR versus yes: 1.83, 95%CI: 1.45–2.31) or beyond on-site vaccination (aOR versus yes: 1.86, 95%CI: 1.66–2.10), perceived the payment as a little easy (aOR versus very easy: 1.99, 95%CI: 1.71–2.32), and did not have free IV (aOR versus free vaccination: 1.60, 95%CI: 1.42–1.81) (Table 2).

## 7. Main Drivers of Influenza Vaccine Hesitancy in 3Cs’ Model

**Figure 2:**
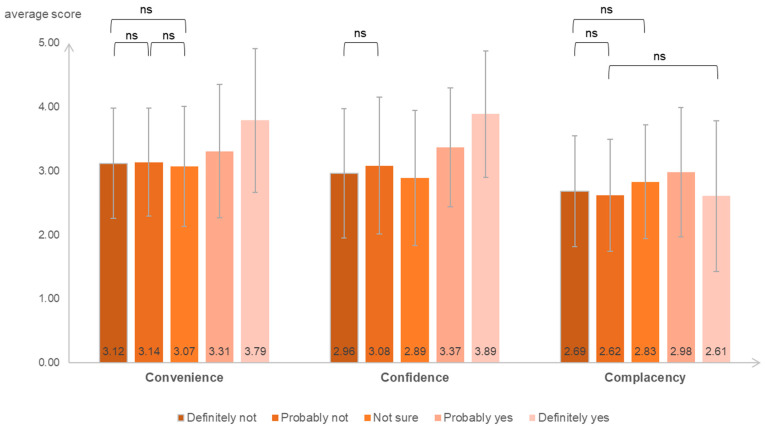
Average scores based on the “3Cs’ model” were categorized by the question about influenza vaccine willingness and the results of multiple comparisons. The WHO used the “3Cs’ model” to classify vaccine hesitancy: confidence, complacency, and convenience, shown at the *x*-axis. The *y*-axis represented the average five-point Likert scale of these three central factors. The differences between answers were significant at 0.01 statistical level unless noted “ns” by a connecting line. ns: not significant.

The average scores in hesitancy, moderate acceptance, and high acceptance groups were: 3.12 ± 0.86, 3.31 ± 1.04, 3.79 ± 1.12 for convenience factor; 3.03 ± 1.06, 3.37 ± 0.93, 3.89 ± 0.99 for confidence factor; and 2.66 ± 0.88, 2.98 ± 1.01, 2.61 ± 1.18 for complacency factor, respectively. Among the three central factors, HCWs reporting high convenience and confidence were less likely to report an increase in IVH, while those with the lowest scores were waverers. However, the result of complacency was irregular: high acceptance held the lowest, while moderate presented the highest complacency. There were significant differences among the acceptance and hesitancy groups, but the differences within the hesitancy group were observed less, as shown in Figure 2. Three responses in the hesitancy group did not show significant convenience, two refusal responses did not have significant confidence, and the differences between direct refusal and moderate refusal or indecisiveness were not statistically significant. 

Spearman’s correlation showed that HCWs’ recommendation willingness had a significant positive correlation with their ability to answer patient questions (r  =  0.307, *p*  <  0.001). Their influenza vaccination willingness and personal perceived risk also showed the same trend (r  =  −0.441, *p*  <  0.001; r  =  −0.237, *p*  <  0.001). However, there was no significant correlation between their recommendation intention and professional title or workplace attitude (r  =  0.013, *p*  =  0.082; r  =  0.013, *p*  =  0.087). Adjusting control variables (influenza vaccination willingness and personal perceived risk) that could affect recommendation willingness, the partial correlation showed that recommendation behaviour was still significantly associated with their ability to answer patient questions (r  =  0.178, *p*  <  0.001). 

**Figure 3:**
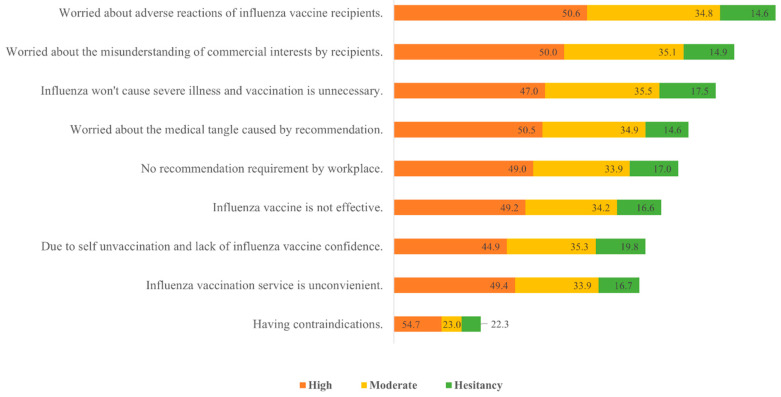
Barriers to influenza vaccine recommendation by healthcare workers (%). The bar chart showed the importance rank by total weight value shown by the length of the bars, and each group percentage of weight value among reasons was presented. Percentages may not total to 100 owing to round.

A multiple-choice ranking question examined impediments to recommendations. Generally, HCWs were concerned about the adverse reactions of the vaccine in their patients. Additionally, they were worried about being misunderstood as having commercial interests and did not accept the need for IV (Figure 3). In the hesitancy group, HCWs showed a greater absence of the belief that IV was necessary and insisted that influenza was a mild disease.

## 8. Discussion

Vaccination protection within HCWs is essential, considering that it could reduce the intensity and spread of the infection and keep the healthcare system robust [14,15]. HCWs are exposed as frontline workers to infectious diseases. Vaccines can protect their occupational health and safety and highlight the duties, rights, and responsibilities for health and safety at work. As reported by a previous study, IVC of HCWs (67%) had substantial growth after the official document released in China, which required all medical institutions to provide IV [16], but dropped dramatically due to the COVID-19 pandemic [6]. Fortunately, we did not observe a decline in IV willingness for the coming season (74.89%) which may attest to the advantage of free IV for HCWs. Although the willingness was desirable in our study, the results are still worrying because nearly half (43.72%) of the HCWs showed only moderate acceptance. The gap between willingness and behaviour is more likely to drive them into a delay or refusal group. Their intention to recommend IV (82.58%), which was higher than the intention to be vaccinated, was also investigated in this study. 

Vaccine hesitancy has become a familiar term, while there is still some unclear usage in research, especially when researchers remained unaware of its reclarification by the Strategic Advisory Group of Experts on Immunization in May [9,17,18]. This term focuses on a psychological process rather than a behavioural expression. In the 3Cs’ model, the relative statistical consistency of HCWs answering with “no” or “not sure” illustrated the definition that covers being both opposed to and confused about IV [17]. Meanwhile, a reference BeSD survey was appealed to be adopted during the investigation of the drivers of vaccination worldwide. Vaccine hesitancy depends on individuals and populations as well as on the vaccines. Thus, an HCW who supports the national program of immunization or COVID-19 vaccine is likely to have IVH. The extent of IVH may vary depending on their characteristics. This survey provided a questionnaire on influenza vaccination for Chinese HCWs. Our questionnaire integrated the BeSD survey for HCWs and found that almost domains adopted from BeSD had significant differences in IVH and IV acceptance groups. 

Regarding the sociodemographic characteristics, differences were observed mainly in the IVH group and rarely in the moderate acceptance group when compared with high acceptance HCWs except for gender. Among Chinese HCWs, IVH was correlated with men, higher economic levels, workplaces beyond hospitals, lower professional titles, and probable development of chronic diseases. Worldwide, the impacting factors shared common personal characteristics but varied in levels within each characteristic due to complicated cultural or conceptual backgrounds [19]. Thus, further studies are needed to that consider the actual domestic scenarios.

Vaccine acceptance is discussed as a psychological topic in research [20,21]. The strongest association found between the thinking and feeling domain and IVH was expected: HCWs belonging to this category (69.74%) were those who considered IV as less important. However, they contributed to the perceived lower-risk category in this survey. This confusing result may be explained by their high rate of willingness in contrast to the widely held perception of influenza as not severe, which encountered limitations of statistical methods. Moreover, the findings indicated that HCWs equipped with IV knowledge could improve their willingness. This means that HCWs increased their comprehensive knowledge of IV and showed a favourable attitude; however, they did not change their stubborn opinion about influenza. Many reviews on influenza vaccination consistently indicated that HCWs were hesitant towards IV on the grounds that they were not at risk of contracting influenza and its severe outcomes [8,22]. It is essential to improve HCWs’ perception of the risk of influenza and IV literacy. Admittedly, it requires a long-term commitment rather than an overnight effort. Meanwhile, contrasting findings from research on IVH indicated that a concrete health threat improved vaccination willingness and behaviour. It is plausible that the COVID-19 pandemic and widespread COVID-19 vaccine campaigns heightened the importance of vaccines that made the acceptance of IV beneficial [23]. Learning from these findings, releasing scientific warning information may deepen the perceived health risks with seasonal influenza on the horizon.

Colleague encouragement and compulsive contexts can be effective in reassuring IVH. A positive social process helps break the information cocoon of anti-vaccination by preventing cognitive and emotional aspects from being collected in hesitancy cliques. Thus, education is probably more effective when peers who are on the positive side of IV are involved. The recommendation attitude presented HCWs’ IV willingness well, though some studies reported that vaccination history had an impact on IV rather than recommendation [24,25]. It is promising to observe whether a relationship between their recommendation and vaccination exists in practical scenarios. Moreover, specific types of vaccines correlated with higher acceptance in our study, which indicated that studies on preferences are needed after the willingness has increased. It is reasonable to assume that gaps between intention and behaviour would be narrowed when the vaccine supply meets the demand according to the preference. 

Both confidence and complacency in the 3Cs’ model predominantly refer to the psychological state [26,27]. It considers the IVH in another category. HCWs with high confidence toward IV and vaccination maintain intensive acceptance. This confidence may be acquired from personal knowledge or feeling about the vaccine and healthcare service or from social processes formed by intimate relationships. For complacency, it was similar to the thinking and feeling domain in the BeSD framework, such as the perceived risk of influenza.

For HCWs, on-site vaccination was more feasible than vaccination in other workplaces. Accordingly, social processes and practical issues related to the workplace played distinct roles when the HCWs chose their conduct. Meanwhile, the practical issues domain could explain the convenience factor in the 3Cs’ model the most. On-site vaccination, easier payment, and free vaccination close the gap between consciousness and action. In fact, some hospitals have not made free and on-site IV available for all HCWs in spite of the request by the National Health Commission, while others introduced a hard policy without caution, leading to negative responses [19]. As an alternative to compulsory vaccination, non-statutory policies, such as the frequency of declaration of health and wearing high-level protective masks, are rendered “inconvenient” by the decline group [28]. More than a quarter of the participants mentioned that they were beyond or unaware of these favourable conditions currently. The truth is that interventions are the basis, and implementation determines performance. Therefore, the evaluation of the process is essential. 

In the BeSD framework, the motivation domain refers to vaccination willingness or intention, which is determined by the thinking, feeling, and social processes domains. As the antecedent of the vaccination domain, they are affected by the practical issues domain. In this study, influenza vaccination willingness was used to predict whether the vaccination was influenced by practical issues. The results, on the one hand, proved that the assumption that considers intention as a substitute for behaviour was rational; on the other hand, it was reasonable to infer that practical issues may have an impact on motivation since some variables may act on more than one domain [9]. Moreover, when a mandatory policy is enacted, bypassing any motivation, these drivers would break pathways through the BeSD framework [21]. For example, when the implementation of applied sanctions or coercive measurement restricts the range of choice, HCWs’ motivation rarely explains their vaccination. However, it remains unclear whether the motivation would remain once these requirements are reversed. 

As a priority group for vaccination, HCWs’ vaccination could benefit both themselves and patients by preventing respiratory infections. Their behaviour and recommendations are among the most powerful influencers in vaccination decisions. The importance of HCWs’ vaccine recommendations in the decision-making process has been well documented, but their awareness, intention, and behaviour in making recommendations lacked research [29]. The results not only verified that HCWs’ intention of IV correlated with their intention of recommendation but also indicated the key to improving it by boosting their capacity to answer patient questions about receiving IV regardless of their work departments. 

Apart from IVH, the top reasons that impeded HCWs’ recommendation included concerns about recipients’ adverse reactions, worry about being misunderstood as having a commercial advantage, and considering influenza as less serious. Separate groups have different reasons. Compared with the groups with positive recommendation intention, the hesitancy group considered influenza less serious. Their intention was further affected by whether they were vaccinated. Thus, suboptimal coverage raises a bigger threat to HCWs and their reach. Therefore, it is of utmost importance to equip HCWs with scientific knowledge and correct perspectives to prevent influenza and promote influenza vaccination. 

The investigation question in the IV survey focused more on HCWs as the general population but ignored their perspective in the context of healthcare systems. It is supposed that HCWs sometimes fail to realize their vulnerability to seasonal influenza and the importance of influenza vaccination due to their unpredictable occupational contexts and professional responsibilities. Further surveys are expected to unveil why HCWs fail to characterize influenza as a significant threat, the kind of adverse reactions they are worrying about, and the perceived importance of their recommendation and model roles for patients to refine education interventions appropriately to the situation.

Although researchers have reported on the willingness of HCWs to receive influenza vaccination, it is essential to ensure that their willingness would not be affected by too many COVID-19 vaccination campaigns this year. Moreover, this study is the first to use the BeSD survey towards IV among HCWs to examine its applicability in China and provide evidence to the world. However, there are some limitations. The observational results were mainly obtained from survey toolkits, which weakens their soundness. However, its conclusions guide the experimental and implementation of research findings specifically to verify and deepen these findings in the future. Collecting data from the platform of respiratory disease care professionals, where the HCWs were more likely from economically developed cities and focused more on influenza, may induce potential selection bias and lead to an overestimation of the coverage rate and willingness. Furthermore, the self-reported assessment with a possibility of recall bias is another limitation. Despite such an effect likely to be minimal considering the latest memory, future assessment is reasonable that provides follow-up data from registration data. Moreover, the results should be extrapolated with caution because some reasons addressed were specific to China.

## 9. Conclusions

In conclusion, HCWs bear the brunt of influenza, and their willingness to vaccination must be clear to guarantee optimal coverage every year. Interventions in a rut are undesirable to achieve the expectations since situations that have an impact on HCWs’ attitudes change each season. In this respect, responses and feedback are advocated to correct HCWs’ misunderstandings, especially those of the participants following the surveys. Specifically, the HCWs who have raised awareness of IV to a certain extent but doubt themselves to be at risk of contracting the influenza infection. 

## Figures and Tables

**Table 1:** Healthcare workers’ characteristics and their attitude toward influenza vaccines (N = 17,832).

Characteristics	Levels	N	%	High Acceptance (n = 7516)		Moderate Acceptance (n = 5839)	
				n	% (95% CI)	n	% (95% CI)
Gender							
	Male	10,042	56.31	3753	37.37 (36.43–38.32)	3338	33.24 (32.32–34.17)
Per capita disposable income ^a^							
	Low	1762	9.88	864	49.04 (46.70–51.37)	593	33.65 (31.48–35.89)
	Moderate	4283	24.02	1940	45.30 (43.81–46.79)	1468	34.28 (32.86–35.71)
	High	11,787	66.10	4712	39.98 (39.09–40.86)	3778	32.05 (31.21–32.90)
Type of workplace							
	Primary hospitals/Community health centers	2977	16.69	1419	47.67 (45.87–49.46)	951	31.94 (30.29–33.64)
	Secondary hospitals	4577	25.67	1923	42.01 (40.59–43.45)	1491	32.58 (31.23–33.94)
	Tertiary hospitals	5979	33.53	2720	45.49 (44.23–46.76)	1930	32.28 (31.10–33.47)
	Medical education or academic institutions	1255	7.04	315	25.10 (22.76–27.55)	471	37.53 (34.88–40.23)
	Centers for disease control and prevention	1614	9.05	634	39.28 (36.92–41.68)	502	31.10 (28.88–33.39)
	Health-related public organizations	1334	7.48	469	35.16 (32.63–37.75)	451	33.81 (31.31–36.38)
	Others	96	0.54	36	37.50 (28.30–47.44)	43	44.79 (35.12–54.77)
Professional title ^b^							
	Senior	5297	29.71	2440	46.06 (44.72–47.41)	1929	36.42 (35.13–37.72)
	Intermediate	5740	32.19	2610	45.47 (44.18–46.76)	1870	32.58 (31.37–33.80)
	Junior	4707	26.40	1565	33.25 (31.91–34.60)	1454	30.89 (29.58–32.22)
	None	2088	11.71	901	43.15 (41.04–45.28)	586	28.07 (26.17–30.02)
Chronic diseases							
	Yes	4778	26.79	1574	32.94 (31.62–34.29)	1450	30.35 (29.06–31.66)
Perceived risk of influenza this season							
	Not at all concerned	6672	37.42	4216	63.19 (62.03–64.34)	1618	24.25 (23.23–25.29)
	A little concerned	7170	40.21	2322	32.38 (31.31–33.48)	3003	41.88 (40.74–43.03)
	Moderately concerned	3171	17.78	659	20.78 (19.40–22.22)	1071	33.77 (32.14–35.44)
	Very concerned	819	4.59	319	38.95 (35.65–42.32)	147	17.95 (15.44–20.69)
Confidence in influenza vaccine benefits							
	Not at all important	1713	9.61	802	46.82 (44.46–49.19)	449	26.21 (24.17–28.33)
	A little important	5610	31.46	1033	18.41 (17.42–19.44)	2736	48.77 (47.46–50.08)
	Moderately important	5113	28.67	1429	27.95 (26.73–29.19)	2010	39.31 (37.98–40.66)
	Very important	5396	30.26	4252	78.80 (77.69–79.87)	644	11.93 (11.09–12.82)
Ability to answer influenza vaccine questions							
	Not at all confident	2676	15.01	927	34.64 (32.86–36.46)	888	33.18 (31.42–34.99)
	A little confident	5280	29.61	1085	20.55 (19.48–21.66)	2267	42.94 (41.60–44.27)
	Moderately confident	4442	24.91	1696	38.18 (36.76–39.62)	1677	37.75 (36.34–39.19)
	Very confident	5434	30.47	3808	70.08 (68.85–71.28)	1007	18.53 (17.52–19.58)
Colleague norms ^c^							
	Yes	10,930	61.29	5335	48.81 (47.87–49.75)	3380	30.92 (30.06–31.80)
	No	3739	20.97	1160	31.02 (29.56–32.52)	1235	33.03 (31.54–34.55)
	Unclear	3163	17.74	1021	32.28 (30.67–33.92)	1224	38.70 (37.01–40.40)
Attitude toward influenza vaccine by workplace this season							
	Required	2788	15.63	1538	55.16 (53.31–57.00)	758	27.19 (25.56–28.86)
	Encouraged	10,918	61.23	4730	43.32 (42.40–44.25)	3622	33.17 (32.30–34.06)
	Neutrality	2945	16.52	826	28.05 (26.45–29.69)	1053	35.76 (34.04–37.50)
	Unclear	1181	6.62	422	35.73 (33.04–38.50)	406	34.38 (31.71–37.12)
Influenza vaccine valent							
	Trivalent	7722	43.30	3443	44.59 (43.48–45.70)	2453	31.77 (30.73–32.81)
	Quadrivalent	6502	36.46	2281	35.08 (33.93–36.25)	2298	35.34 (34.19–36.51)
	Unaffected	3608	20.23	1792	49.67 (48.04–51.30)	1088	30.16 (28.67–31.67)
Influenza vaccine type							
	Inactive vaccine	9036	50.67	4318	47.79 (46.76–48.82)	2980	32.98 (32.02–33.95)
	live attenuated vaccine	5380	30.17	1472	27.36 (26.18–28.56)	1872	34.80 (33.53–36.08)
	Unaffected	3416	19.16	1726	50.53 (48.85–52.20)	987	28.89 (27.39–30.43)
Recommendation willingness							
	High	8562	48.01	5519	64.46 (63.44–65.47)	1965	22.95 (22.07–23.85)
	Moderate	6164	34.57	1469	23.83 (22.78–24.91)	2878	46.69 (45.45–47.94)
	Hesitancy	3106	17.42	528	17.00 (15.71–18.35)	996	32.07 (30.44–33.72)
2021/2022 influenza vaccination site							
	Hospital	4766	26.73	2765	58.02 (56.61–59.41)	1306	27.40 (26.15–28.68)
	Centers for disease control and prevention	4719	26.46	1740	36.87 (35.50–38.26)	1553	32.91 (31.58–34.26)
	Community health centers	2600	14.58	1112	42.77 (40.88–44.68)	742	28.54 (26.83–30.30)
	Others	12	0.07	8	66.67 (38.76–87.55)	1	8.33 (0.91–32.85)
	No	5735	32.16	1891	32.97 (31.77–34.20)	2237	39.01 (37.75–40.27)
On-site vaccination							
	Yes	12,871	72.18	6408	49.79 (48.92–50.65)	4122	32.03 (31.22–32.84)
	No	4166	23.36	922	22.13 (20.89–23.41)	1411	33.87 (32.44–35.32)
	Not sure	795	4.46	186	23.40 (20.55–26.43)	306	38.49 (35.16–41.91)
Ways of influenza vaccine payment							
	Self-paid	4557	25.56	2059	45.18 (43.74–46.63)	1450	31.82 (30.48–33.18)
	Free	7229	40.54	3026	41.86 (40.73–43.00)	2278	31.51 (30.45–32.59)
	Employer paid	3385	18.98	1242	36.69 (35.08–38.33)	1134	33.50 (31.93–35.10)
	Medical insurance	2587	14.51	1156	44.68 (42.78–46.61)	962	37.19 (35.34–39.06)
	Others	74	0.41	33	44.59 (33.66–55.95)	15	20.27 (12.35–30.46)
Affordability ^d^							
	Not at all easy	1875	10.51	660	35.20 (33.06–37.38)	591	31.52 (29.45–33.65)
	A little easy	4824	27.05	1058	21.93 (20.78–23.12)	1891	39.20 (37.83–40.58)
	Moderately easy	6080	34.10	2166	35.63 (34.43–36.84)	2477	40.74 (39.51–41.98)
	Very easy	5053	28.34	3632	71.88 (70.63–73.10)	880	17.42 (16.39–18.48)
Free vaccination provided by employers							
	Yes	11,195	62.78	5777	51.60 (50.68–52.53)	3435	30.68 (29.83–31.54)
	No	4558	25.56	1106	24.27 (23.04–25.53)	1604	35.19 (33.81–36.59)
	Unclear	2079	11.66	633	30.45 (28.50–32.45)	800	38.48 (36.41–40.59)

Percentages may not total 100 owing to rounding. ^a^: In terms of per capita disposable income, provinces are divided into three levels: low, moderate, and high. Low for Jilin, Shanxi, Heilongjiang, Henan, Guangxi, Xinjiang, Qinghai, Guizhou, Tibet, Yunnan, and Gansu; moderate for Inner Mongolia, Chongqing, Hunan, Anhui, Hubei, Jiangxi, Shaanxi, Hainan, Hebei, Sichuan, Ningxia; high for Beijing, Shanghai, Tianjin, Jiangsu, Zhejiang, Fujian, Guangdong, Shandong, Liaoning, Hongkong, Taiwan. ^b^: Junior equals a resident physician; intermediate equals an attending physician; senior equals a chief physician. ^c^: College norms were asked by the question, “Do you think most of the people you work with will get an influenza vaccine?” to assess descriptive social norms at the workplace. “Most people you work with” includes all colleagues and people at their place of work who could be eligible for an influenza vaccine. ^d^: The affordability item assessed the perceived cost of vaccination, including not only the cost of vaccination but the cost of traveling to the vaccination site plus the cost of taking time away from work.

**Table 2:** Factors associated with influenza vaccine willingness-multivariable logistic regression analysis (reference: high acceptance group).

		Hesitancy		Moderate Acceptance	
Characteristics	Levels	aOR 95%CI	*p*.Value	aOR 95%CI	*p*.Value
Gender					
	Female	ref		ref	
	Male	1.41 (1.28–1.56)	**<0.01**	1.24 (1.14–1.36)	**<0.01**
Per capita disposable income					
	High	ref		ref	
	Moderate	0.83 (0.74–0.94)	**<0.01**	1.00 (0.90–1.10)	0.93
	Low	0.70 (0.59–0.83)	**<0.01**	0.92 (0.80–1.06)	0.24
Hospital level					
	Tertiary	ref		ref	
	Secondary	1.02 (0.90–1.15)	0.78	0.95 (0.85–1.06)	0.34
	Primary/Community health centers	0.98 (0.84–1.14)	0.78	0.96 (0.85–1.09)	0.55
	non-nosocomial	1.20 (1.05–1.36)	**0.01**	1.01 (0.90–1.13)	0.85
Professional title					
	Senior	ref		ref	
	Intermediate	1.27 (1.11–1.44)	**<0.01**	1.00 (0.90–1.11)	0.98
	Junior	1.82 (1.57–2.10)	**<0.01**	1.08 (0.95–1.22)	0.24
	None	1.40 (1.18–1.67)	**<0.01**	0.90 (0.77–1.05)	0.18
Chronic diseases					
	No	ref		ref	
	Yes/Unclear	1.58 (1.41–1.77)	**<0.01**	1.02 (0.92–1.13)	0.76
Thinking and feeling					
Confidence in influenza vaccine benefits					
	Very important	ref		ref	
	Moderately important	3.93 (3.41–4.53)	**<0.01**	4.55 (4.04–5.12)	**<0.01**
	A little important	4.69 (4.04–5.43)	**<0.01**	7.26 (6.42–8.22)	**<0.01**
	Not at all important	1.97 (1.63–2.37)	**<0.01**	2.27 (1.93–2.69)	**<0.01**
Perceived risk of influenza					
	Very concerned	ref		ref	
	Moderately concerned	0.84 (0.66–1.06)	0.13	1.37 (1.07–1.76)	**0.01**
	A little concerned	0.36 (0.29–0.45)	**<0.01**	1.10 (0.86–1.39)	0.45
	Not at all concerned	0.24 (0.19–0.30)	**<0.01**	0.71 (0.56–0.90)	**<0.01**
Ability to answer questions					
	Very confident	ref		ref	
	Moderately confident	1.44 (1.25–1.67)	**<0.01**	1.49 (1.33–1.68)	**<0.01**
	A little confident	2.13 (1.85–2.46)	**<0.01**	1.99 (1.76–2.25)	**<0.01**
	Not at all confident	1.63 (1.39–1.91)	**<0.01**	1.46 (1.27–1.67)	**<0.01**
Social processes					
Colleague norms					
	Yes	ref		ref	
	No	1.57 (1.39–1.78)	**<0.01**	1.13 (1.00–1.26)	**0.04**
	Unclear	1.39 (1.19–1.62)	**<0.01**	1.27 (1.11–1.45)	**<0.01**
Attitude toward influenza vaccine by workplace this season					
	Required	ref		ref	
	Encouraged	1.15 (1.00–1.33)	0.06	1.20 (1.07–1.35)	**<0.01**
	Others (Neutrality/Unclear)	1.19 (1.00–1.42)	**0.04**	0.99 (0.85–1.15)	0.89
Motivation					
Influenza vaccine valent					
	Trivalent	0.79 (0.67–0.94)	**0.01**	0.89 (0.77–1.04)	0.14
	quadrivalent	0.88 (0.75–1.05)	0.15	0.98 (0.85–1.13)	0.78
	Unaffected	ref		ref	
Influenza vaccine type					
	Inactive vaccine	1.11 (0.94–1.31)	0.23	1.34 (1.16–1.55)	**<0.01**
	live attenuated vaccine	2.05 (1.72–2.44)	**<0.01**	1.60 (1.37–1.87)	**<0.01**
	Unaffected	ref		ref	
Willingness to recommend					
	High	ref		ref	
	Moderate	2.64 (2.36–2.96)	**<0.01**	2.54 (2.31–2.80)	**<0.01**
	Hesitancy	5.31 (4.60–6.13)	**<0.01**	2.55 (2.22–2.94)	**<0.01**
Practical issues					
2021/2022 influenza vaccination					
	Yes	ref		ref	
	No	1.28 (1.15–1.44)	**<0.01**	1.28 (1.16–1.41)	**<0.01**
On-site vaccination					
	Yes	ref		ref	
	No	1.86 (1.66–2.10)	**<0.01**	1.18 (1.06–1.32)	**<0.01**
	Not sure	1.83 (1.45–2.31)	**<0.01**	1.23 (0.99–1.54)	0.06
Affordability					
	Very easy	ref		ref	
	Moderately easy	1.42 (1.23–1.63)	**<0.01**	1.69 (1.51–1.89)	**<0.01**
	A little easy	1.99 (1.71–2.32)	**<0.01**	1.71 (1.50–1.95)	**<0.01**
	Not at all easy	1.71 (1.42–2.05)	**<0.01**	1.48 (1.25–1.74)	**<0.01**
Free vaccination provided by employers					
	Yes	ref		ref	
	No	1.60 (1.42–1.81)	**<0.01**	1.11 (0.99–1.23)	0.08
	Unclear	1.40 (1.17–1.67)	**<0.01**	1.11 (0.95–1.30)	0.18

Bolded text indicates statistically significant (*p*.values < 0.05).

## Data Availability

Anonymized individual-level data and datasets generated or analyzed during the current study are available for researchers interested in similar studies. Please contact Luzhao Feng (fengluzhao@cams.cn) and Zhongong Yang (yangweizhong@cams.cn).
